# Japanese adolescents’ time use: The role of household income and parental education

**DOI:** 10.4054/demres.2021.44.9

**Published:** 2021-02-04

**Authors:** Ekaterina Hertog, Muzhi Zhou

**Affiliations:** 1 Department of Sociology, University of Oxford, UK.; 2 Department of Sociology, University of Oxford, UK.

## Abstract

**BACKGROUND:**

How children spend their day is closely linked to their social and developmental outcomes. Children’s time use is associated with their parents’ educational and economic capital, making time use a potential reproduction channel for socioeconomic inequalities.

**OBJECTIVE:**

We evaluate the correlation of natal-family economic resources, parents’ education, and children’s daily time use in Japan.

**METHODS:**

Analysing data from a 2006 Japanese time use survey, we use natal-family income, parental education, and the interaction between them to predict in-school and afterschool study time, leisure time, and sleep time for children aged 10–18.

**RESULTS:**

Children from families with higher incomes and more-educated parents spend a longer time studying after school and less time on sleep and leisure. Parental income and mothers’ and fathers’ education are all independently associated with children’s daily patterns.

**CONCLUSION:**

Our findings suggest that available resources and parental education are important in shaping children’s daily routines and, through these routines, their eventual socioeconomic outcomes.

**CONTRIBUTION:**

This is the first article to simultaneously assess the impact of income and parental education on children’s study, leisure, and sleep time. It is also the first paper to analyse children’s time use and their natal-family characteristics in Japan.

## 1. Introduction

Do children from families of different socioeconomic status spend their time differently? Can some of the well-established educational performance gap across socioeconomic groups (Crawford et al. 2017; Jæger and Breen 2016; Putnam 2015) be explained by the fact that children from richer families spend more time studying? And if wealthier children spend more time studying, do they spend less on leisure and sleep?

How children spend their time is associated with a range of developmental outcomes. For example, increased learning time translates into better educational achievement, study skills, and socio-emotional development (Andersen, Humlum, and Nandrup 2016; Kidron and Lindsay 2014). However, spending a long time on homework and extracurricular study has also been linked to reduced socio-psychological well-being (Hu and Mu 2020). Children having adequate sleep is critical to their health, well-being, educational performance, and non-cognitive development (see Hofferth and Sandberg 2001; Alfonsi et al. 2020; Wheaton, Chapman, and Croft 2016).

Research on children’s time use has primarily been conducted in European and Anglophone contexts, and children’s time spent on developmentally beneficial activities has been the main focus. For example, Spanish and British children of better-educated parents spend more time on developmentally enriching activities (including study time) with their parents (Gracia and Garcia-Roman 2018; Mullan 2009).

Less is known about the way natal-family characteristics are associated with other activities, especially children’s time spent on leisure activities and sleep (a few exceptions include Gracia et al. 2019; Hawkins and Takeuchi 2016; Holstein et al. 2019). It also remains unclear whether these educational gradients reflect variation in parenting approaches among parents with different levels of educational attainment, or whether they are instead a proxy for family economic resources (e.g., Doepke and Zilibotti 2019; Lareau 2003, 2011).

We provide a picture of children’s time-use patterns across families of varied income and educational status in Japan, where time-use research on children is scarce. We analyse whether household income and parental education are associated with children’s daily time-use patterns independently of each other or whether the two interact with each other.

Being characterised as a ‘mass-education society’ with nationwide educational zeal, Japan is distinct from its Western or Anglo-Saxon counterparts: In 2018, 60% of Japanese aged between 25 to 34 had tertiary-level education, while the OECD average was 44% (OECD 2020). Japanese society is also characterised by its glorification of effort (Yamamoto and Satoh 2019). A famous saying encouraging school children to study can be translated as “sleep for four hours a day and succeed, sleep for five hours a day and fail”. Given the overall high levels of educational attainment in Japan and the widespread social norms promoting studying effort and stressing that sleep should be sacrificed for studying, one would expect little variation by natal-family background.

## 2. Data, measures, and methods

We analyse data from the 7^th^ wave of the Japanese Survey on Time Use and Leisure Activities (STULA). It is a nationally representative time-use survey which collected the pre-coded activities of 175,000 individuals every 15 minutes over two consecutive days in 2006. The two days were randomly chosen by the Statistical Bureau staff over nine days in mid-October 2006, with the weekends oversampled. The survey is household-based and also records basic sociodemographic information on all household members aged 10 or more, thereby enabling analysis of the association between children’s time use and natal-family characteristics.

We selected children aged 10 to 18 coresiding with both parents and currently in education. We excluded children living in single parent families (8% of the sample), as we are interested in examining the relative importance of both maternal and paternal characteristics, especially education. Very few children in the selected age group are not in school. Education is compulsory until the age of 15 in Japan, and from 15 to 18 children can attend high schools. In 2005, 98% of all Japanese children of high school age attended high schools (MEXT 2017).

The final sample consists of 24,518 diaries collected from 12,290 individuals. Forty-eight per cent of the respondents were female and 52% were male. Over 99% of the respondents completed time diaries on two consecutive days.

Our outcome variables are the number of minutes spent on (1) studying at school, (2) studying outside school, (3) leisure, and (4) sleep. The data only include a generic measure of ‘study time’, defined as studying at school, homework, and extracurricular studying. We use the information on the time of day and day of the week to distinguish between studying at school and studying outside the school setting. We treat study time reported on weekdays between 8:30 and 16:00 as studying at school and study time reported after 16:00 on weekdays and on weekends as out-of-school study. The ‘leisure time’ variable combines the following activities: sport time, leisure time, volunteering, socialising, and media consumption.

Our key predictors include logged household income, and the combined educational levels of the parents. Household income is transformed from the original categorical income variable into a continuous variable using midpoints for the 12 categories provided and then logged. We measure fathers’ and mothers’ education levels separately using categorical variables with 3 categories: high school education or less (55% of fathers and 57% of mothers), college or professional school education (8% of fathers and 31% of mothers), and university education (37% of fathers and 12% of mothers). University education means at least a bachelor’s degree from a 4-year university course.

We control for the child’s gender, current school level, and number of siblings younger than 20 in the household; whether the household includes co-residing grandparents; whether the respondent lives in one of the large urban conglomerates; whether the diary day is a weekend or a weekday; and mother’s and father’s time availability. A child’s school level is used as a proxy for their age because single-year age information is not available. This variable has three categories: primary school (aged 10 to 12), junior high school (aged 13 to 15), and high school (aged 16 to 18). Given the Japanese culture of long working hours, we proxy parental time availability through their working status and typical work hours: not working, working part-time, working fulltime, and typically working overtime.

Throughout the analysis, we use the original survey weights. These are combined weights provided by the Statistics Bureau Japan to correct for the type of day sampled and sampled population bias.

## 3. Analysis results

**Figure 1:**
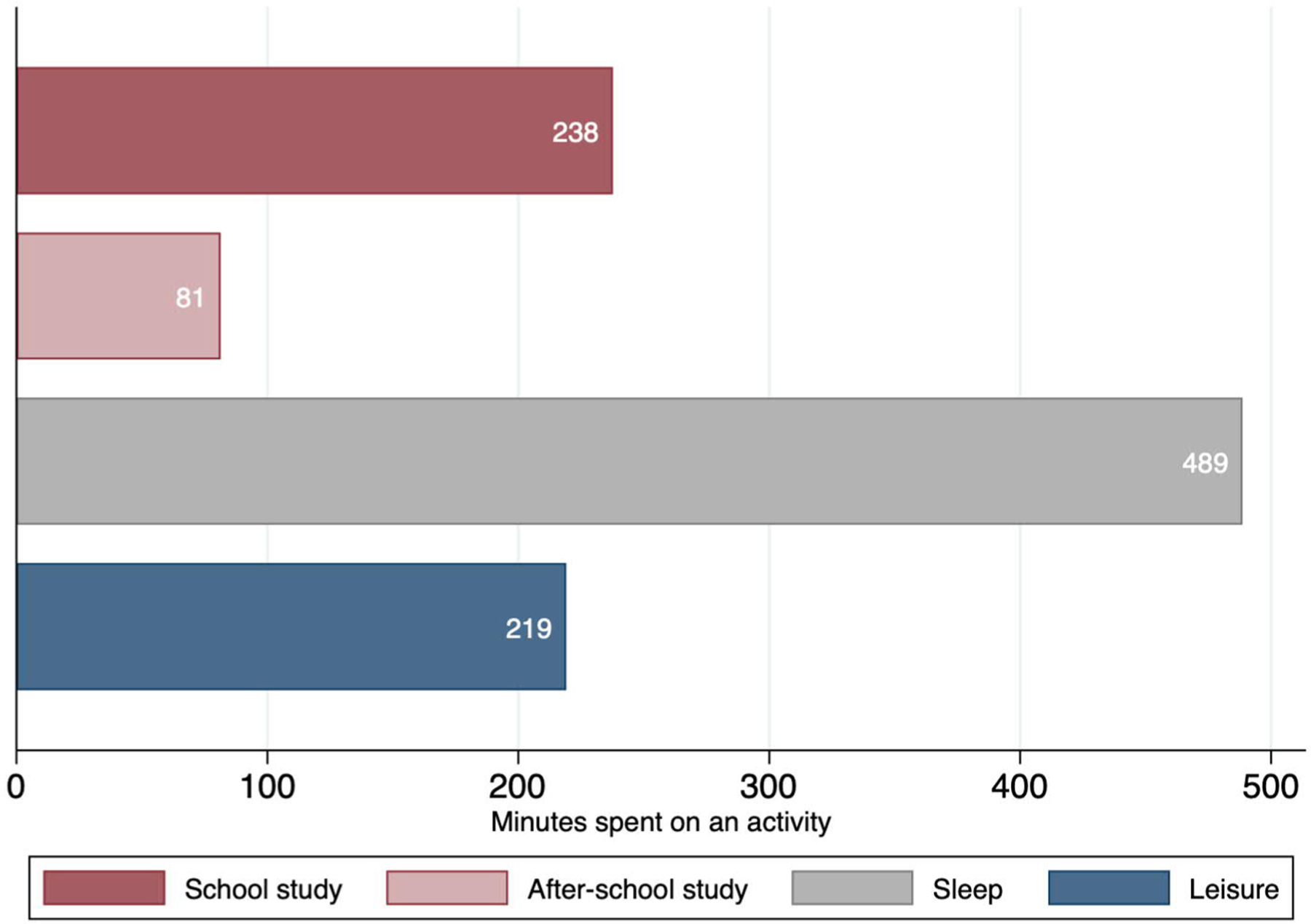
Weighted average daily time spent on studying, sleep, and leisure by Japanese children aged 10 to 18

Figure 1 presents the weighted mean of the minutes spent on study at school, study outside school, sleep, and leisure activities. On average, Japanese adolescents spend over 8 hours per day sleeping, 3 hours and 47 minutes studying during school time, and 3.7 hours on leisure activities.

**Figure 2:**
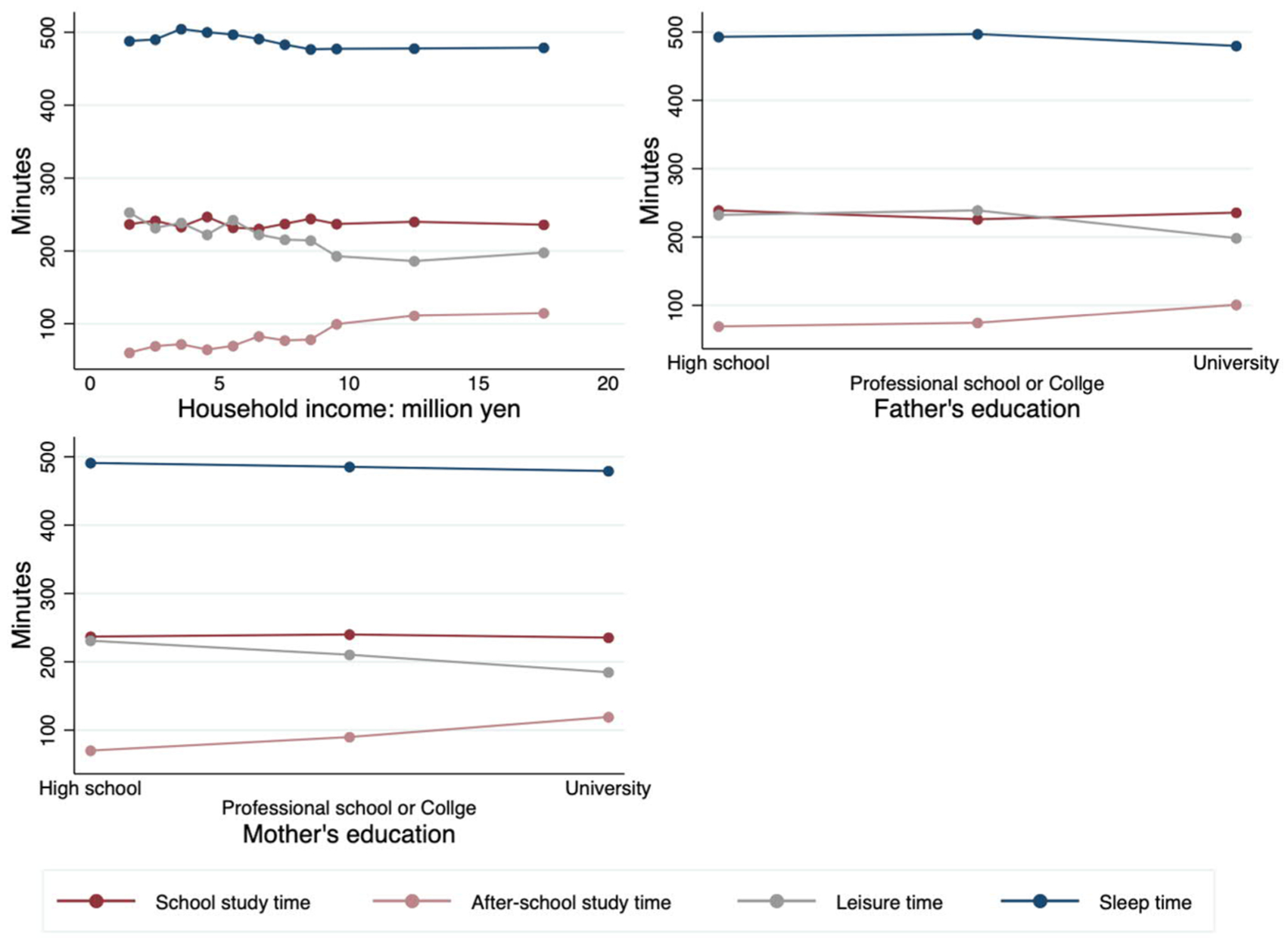
Average daily time children spend on study, sleep, and leisure by natal-family characteristics

Figure 2 breaks down the average minutes children spend on school study, out-of-school study, leisure, and sleep, by different natal-family characteristics. Children from households with higher incomes sleep less, have less leisure time, and spend more time studying outside of school. The more educated the parents, the less time children spend on sleep and leisure and the more time they spend studying.

**Figure 3:**
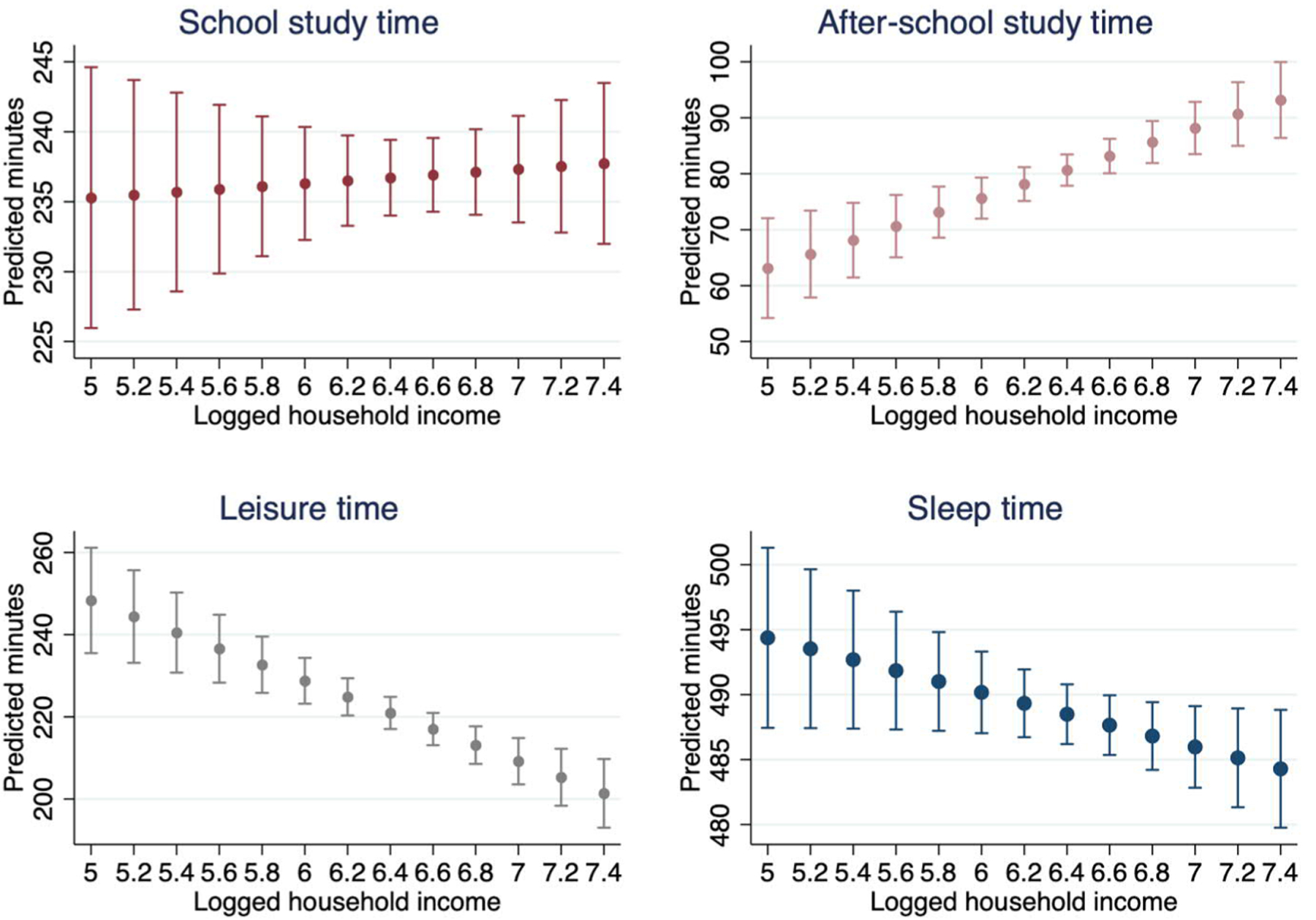
Household income (measured in 10,000 yen per year) and predicted children’s daily time use

**Figure 4:**
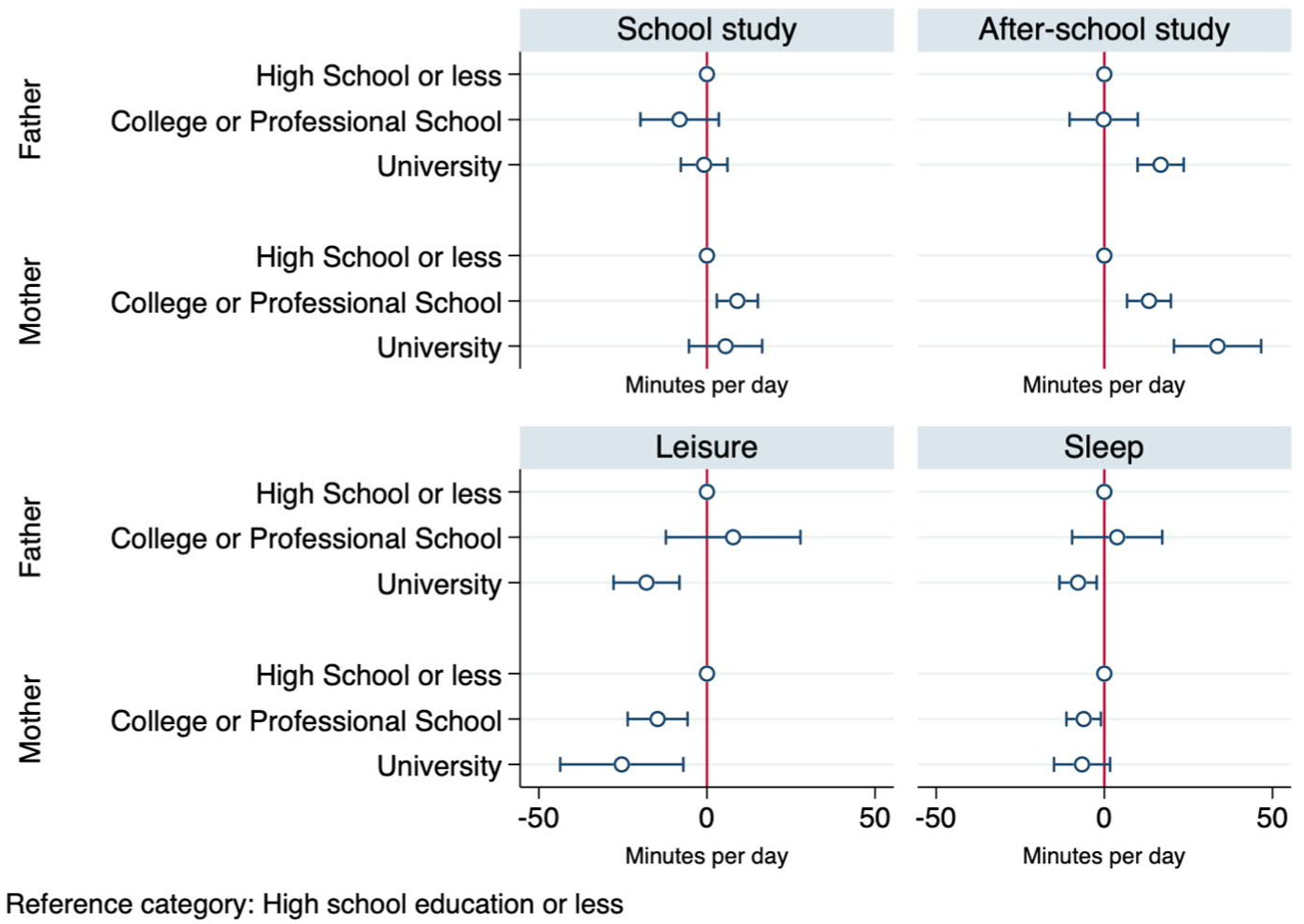
Point estimation with 95% confidence interval of fathers’ and mother’s education in predicting children’s daily time use

**Figure 5:**
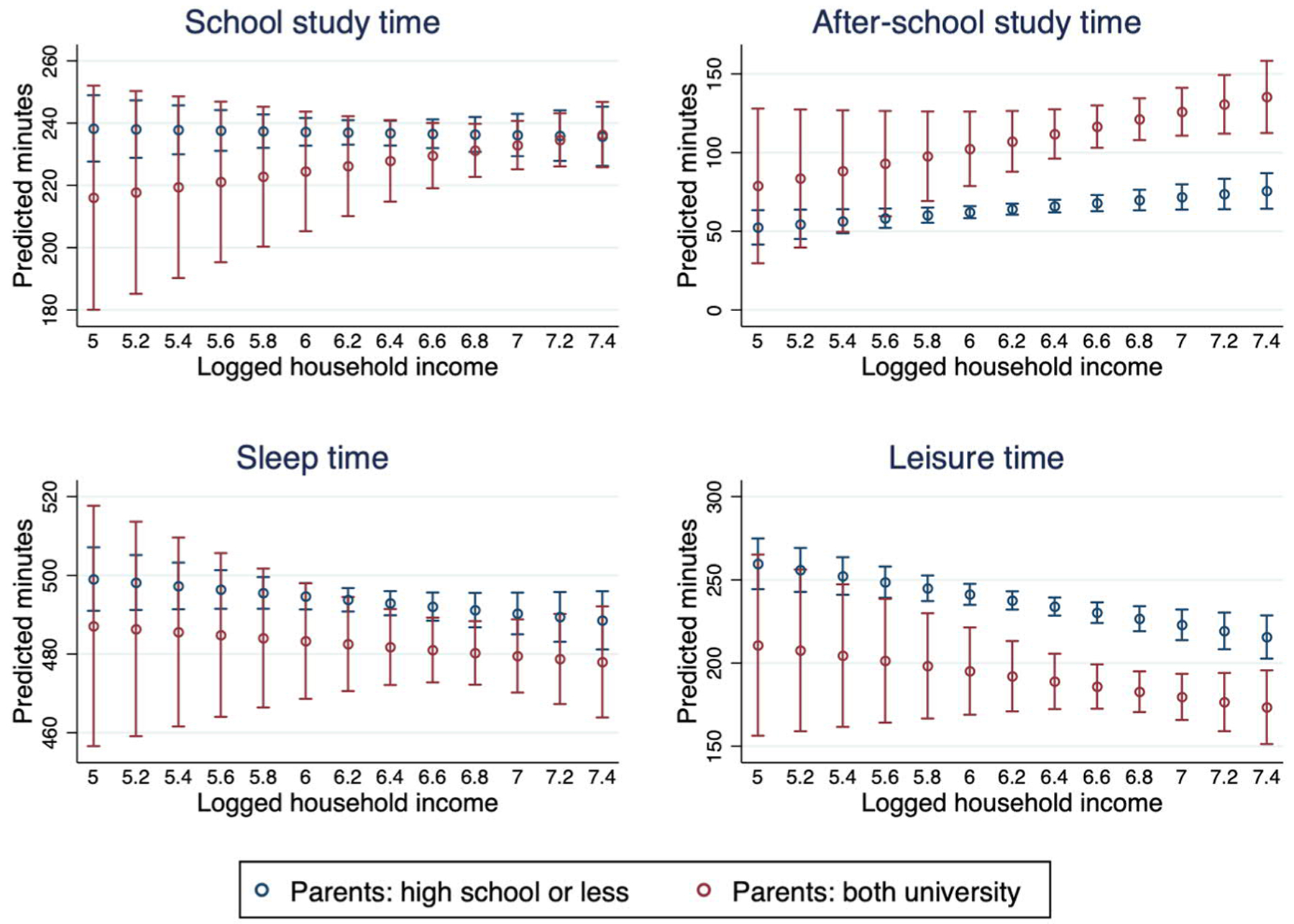
Household income, parents’ education, and children’s daily time use

Most of the above descriptive findings hold in OLS regressions in which we include parental income, father’s and mother’s education, and key control variables in order to predict children’s study, leisure, and sleep times. We present the predicted time-use outcomes in Figures 3 to 5, with other key predictors and control variables set to their mean values.

Figure 3 confirms large differences in children’s daily routines by household income. School study time is not significantly correlated with income, but sleep time is, albeit with some statistical noise. The patterns are clearer for studying outside school and leisure time. A child from the lowest income group is predicted to spend 60 minutes per day studying outside school, whereas a child from the highest income group with otherwise identical family features is predicted to spend 94 minutes. Similarly, a child from a household in the lowest income category is predicted to spend 250 minutes on leisure daily, whereas a child residing in an otherwise identical household in the highest income category is predicted to spend 50 minutes less on leisure daily.

Figure 4 below shows how children’s time spent on daily activities varies with parental education. As in Figure 3, we do not observe significant differences in school study time by parental education, with the exception of a small increase in families where the mother graduated from a professional school or college. Other variables being the same, children in families where the father has a university education or the mother has a tertiary education spend more time studying outside school and less time on sleep and leisure. Notably, children in families where the mother has some tertiary education (attended a college or a professional school) are located between children whose mothers have no tertiary education and children whose mothers attended university in terms of the time they spent studying outside school and the time they have for leisure. By contrast, when it comes to the association between father’s education and children’s time use the only observable difference is between families where fathers have a university education and all other families. The Wald test results also confirm that mother’s college education has a stronger association with children’s time in school, afterschool study time, and leisure than father’s college education. When considering university education, the Wald test results show that the association between mother’s university education and children’s afterschool study time is stronger than the association between father’s university education and children’s afterschool study time. Some of these differences between the associations with mother’s and father’s education could be attributed to the fact that women and men are selected into different types of education. In particular, considerably fewer mothers than fathers in our sample graduated from universities. Consequently, compared to fathers with university education, mothers with university degrees are a more selective group and may care especially strongly about their children’s education. In addition, many mothers who value education strongly may not have managed to attend university themselves and ended up attending colleges or professional schools. Fathers with a similar educational outlook are more likely to be university graduates. This selection effect may be behind the observed greater association between mothers’ college and professional school education and time spent by children on study and leisure.

Could children’s daily routine depend on household income and parental education? Better-educated parents may well be more motivated and skillful in allocating income for activities that could prompt children’s academic effort, e.g., private tutoring. To answer this question we interacted household income and the parental education variable, a combined variable with 9 categories representing all the possible combinations of parental education. To make a clear comparison, we only present the educational groups at the two ends of the spectrum, which are ‘both parents have high school education or less’ and ‘both parents have university education’. The combined results are summarized in Figure 5.

Few families where both parents have not progressed beyond high school in their education have a high income, which explains the large and overlapping confidence intervals at the lower end of the income distribution. We find no clear difference in the income gradients between families with more-educated and less-educated parents. This null finding is confirmed in regression models, where the interaction term for household income and parental education is insignificant for all the activities.

Although they not our main focus, a few findings on the control variables are noteworthy: Japanese children spend more time studying and less time sleeping or doing leisure activities when they progress to higher levels of education. This likely reflects the high-pressure environment, with an emphasis on studying in higher school years. There is no difference in time spent on study, sleep, or leisure between boys and girls. This is unexpected, given the large gender differences in time use between adult Japanese men and women, suggesting that gender differences in time use emerge later in life. Having a sibling is associated with less time spent studying and more time spent on leisure daily. There is no clear pattern in the variation in children’s time use associated with parental time availability. This suggests that, at least for children aged 10 or older, having a non-working parent or living in a family where both parents work is not correlated with the children having different daily routines.

## 4. Discussion and conclusions

This study offers the first time-diary analysis of children’s and adolescents’ major daily activities in Japan and considers their association with household income and parental education. We find that children and adolescents from higher-income households or from households with highly educated parents spend more time studying outside school. This longer study time pattern echoes findings from the United Kingdom, Spain, and Finland, where children of better-educated mothers are found to spend more time studying. The findings on sleep time patterns differ from what we see in other societies. A recent study found no association between maternal education and sleep time in the United Kingdom, Spain, and Finland (Gracia et al. 2019). We find that children from families where a father or both parents have a university education sleep less than children in families where neither of the parents attended university. Finally, household income and parental education are both associated with less leisure time for children in Japan. We do not find evidence supporting an intuition that parental education may enable families to allocate finances to more efficiently encourage desirable behaviours.

Japan is a society characterised by high levels of educational attainment: high school education is virtually universal and the majority of young men and women these days have a tertiary education. Yet even in Japan, parental resources in terms of both economic capital and both parents’ educational attainment seem to shape the amount of time adolescents invest in studying. This finding suggests that the advantages that parental status confers in terms of access to better schools, private tutoring, more books, and other resources at home are compounded by children’s own efforts. This is likely to have serious implications for the disparity in children’s future life chances.

The reduced sleep time among children in families with highly educated parents is also worrying. Research has shown that reduced sleep time is associated with children’s increased risk of obesity and behaviour problems (Sadeh, Gruber, and Raviv 2002; Sivertsen et al. 2014). The impact of reduced leisure time on children’s development is highly dependent on the type of leisure activity. For instance, sedentary leisure activities such as watching TV and gaming are known to be correlated with obesity and short-sightedness (Lissak 2018), while reading and sports are associated with better socio-psychological well-being and (in the case of reading) with better educational outcomes (Hu and Mu 2020). More research is needed to understand both the socioeconomic correlates of leisure time use and their long-term consequences.
